# HarmGR13 mediates myo-inositol taste perception in *Helicoverpa armigera* larvae

**DOI:** 10.1371/journal.pgen.1011744

**Published:** 2025-06-03

**Authors:** Yu-Sheng Tan, Bao-Tong Mo, Guo-Cheng Li, Yu-Ruo Guo, Jian-Zhen Zhang, Chen-Zhu Wang

**Affiliations:** 1 State Key Laboratory of Animal Biodiversity Conservation and Integrated Pest Management, Institute of Zoology, Chinese Academy of Sciences, Beijing, PR China; 2 CAS Center for Excellence in Biotic Interactions, University of Chinese Academy of Sciences, Beijing, PR China; 3 School of Synthetic Biology, Shanxi University, Taiyuan, PR China; National Institute on Deafness and Other Communication Disorders, National Institutes of Health, UNITED STATES OF AMERICA

## Abstract

Myo-inositol, a sugar alcohol produced by most plants, serves as a nutrient and feeding stimulant for many phytophagous insects. Inositol-sensitive taste sensilla have been characterized in many Lepidoptera larvae, but their molecular bases remain unclear. In this study, we determined the gustatory receptors (GRs) for myo-inositol in larva of *Helicoverpa armigera*, a worldwide crop pest. First, electrophysiological analyses revealed that medial sensilla styloconica strongly responded to myo-inositol and ribose, with weaker responses to xylose, and one GRN inside sensillum may mediate the response to these three chemicals. Based on phylogenetic analysis of sugar GRs of Lepidoptera insects and previous results on *Bombyx mori*, we then selected two candidate GRs, HarmGR13 and HarmGR11. Using CRISPR-Cas9, we generated knockout mutants for the two GR genes. Knocking out *HarmGR13* abolished the responses of the sensilla to myo-inositol, ribose, and xylose, while knocking out *HarmGR11* showed no changes. Behavioral assays confirmed that larvae of *HarmGR13* homozygous mutant lost the feeding preference to myo-inositol which the wild-type larvae had. Further functional analysis with *Xenopus* oocytes expressing system and two-electrode voltage-clamping demonstrated that myo-inositol and ribose specifically induced concentration-dependent currents in HarmGR13-expressing oocytes. Structural predictions and molecular docking of HarmGR13 revealed three amino acid residues might be involved in ligand binding. Mutation of these residues resulted in loss of oocyte responses to myo-inositol and ribose. We reveal that HarmGR13 is a receptor that mediates the activity of the cells sensitive to inositol and ribose in larvae, providing new molecular targets for the strategy of regulating the feeding behavior of pests by modifying taste.

## Author summary

The larvae of lepidopteran insects primarily feed on plants, and their sense of taste is mediated by sensilla located on their mouthparts. Here we investigate cellular and molecular mechanisms by which the cotton bollworm larvae perceive inositol, a nutrient widely present in plants. First, we confirmed that a pair of sensilla on the mouthparts were very sensitive to inositol and ribose, and one of the gustatory sensory neurons inside might be responsible for detecting them. Next, we selected two candidate gustatory receptors, HarmGR11 and HarmGR13 to analyze their function through gene editing. We found that the larvae with *HarmGR13* knocked out lost physiological responses to inositol and ribose, while the larvae with *HarmGR11* knocked out responded to inositol in the same way as the wild-type. Ectopic expression further confirmed that the *Xenopus* oocytes expressing HarmGR13 responded to inositol and ribose. Structure prediction and molecular docking suggested three amino acid residues were involved in ligand-receptor binding, and then the subsequent point mutations showed that changing the three residues to nonpolar residues respectively eliminated the tuning activity of HarmGR13. In conclusion, HarmGR13 is an inositol receptor that mediates the physiological and behavioral responses of the larvae to inositol.

## Introduction

Myo-inositol is a ubiquitous sugar alcohol in green plants. It is a key structural component of phospholipids, and it is involved in plant growth and development in many ways, such as cell wall formation, osmoregulation, cell signaling and phosphate storage. It is also a nutrient and energy substance for insects [1,2]. The level of inositol in plants may reflect some important characteristics and nutritional value of plants, and thus become an important compound for insects to recognize host plants and evaluate plant nutritional value.

When an insect comes into contact with a potential host plant, whether to accept or reject it depends largely on its contact chemoreception. Taste sensilla in insects occur outside the mouthparts, and are also often present on the tarsi and other parts of the body [3]. Commonly there are four or six gustatory sensory neurons (GSNs) associated with a single sensilla and their dendrites run through the dendrite sheath, ending just inside the pore. The sensory properties of GSNs are determined by gustatory receptors (GRs) expressed in the dendrite membranes [4]. GRs are seven-transmembrane-domain membrane proteins that bind to tastants and trigger action potentials of neurons. Research on insect GRs began with *Drosophila melanogaster* [5]. Phylogenetic analyses have categorized GRs into four clades, the DmGr43a, sugar receptors, carbon dioxide receptors, and bitter receptors. Except for the carbon dioxide receptors, which are associated with olfactory sensing, the receptors in the other three clades are involved in gustatory sensing. In *Drosophila*, DmGr43a is a fructose receptor only expressed in the gut and certain parts of the brain in adults but primarily expressed in the periphery of larvae [6,7]; eight receptors (DmGr5a, DmGr61a-f and DmGr64f) in the sugar receptor clade are required for adult responses to multiple sugars [8–12]; 33 receptors co-expressed with Gr66a in the bitter receptor clade mediate the detection of bitter substances.

Taste perception of insects for inositol is mostly studied on Lepidopteran insects. Behavioral and electrophysiological studies have shown that caterpillars of many species respond to inositol [13]. Most electrophysiological works have focused on two sensilla on the maxillary galea, the medial sensillum styloconicum and the lateral sensillum styloconicum, which play an important role in host plant recognition [14]. Of the two sensilla, at least one is sensitive to inositol [15]. However, the molecular mechanisms underlying inositol detection by these sensilla remain poorly understood, with only two conflicting reports on *Bombyx mori* available. One study reported that the Sf9 cells expressing the sugar receptor BmGr8 showed concentration-dependent responses to inositol and epi-inositol [16]. In contrast, using the *Xenopus laevis* oocyte expression system and the HEK293T cell expression system, another study found that the cells expressing BmGr10 responded to inositol and epi-inositol, while the cells expressing BmGr8 did not [17]. In either study, the function of the relevant Grs was not validated *in vivo*.

*Helicoverpa armigera* (Lepidoptera: Noctuidae) is a globally significant agricultural pest, causing annual economic losses exceeding $3 billion [18,19]. With a broad host range of over 300 plant species from 68 families, the larvae primarily feed on plant reproductive organs. Previous studies showed that the lateral sensilla styloconica of *H. armigera* larvae were sensitive to sucrose, while the medial sensilla styloconica of *H. armigera* larvae were sensitive to inositol [20]. In a recent study, we showed that the *Xenopus* oocytes expressing *HarmGR10* highly expressed in the maxillary galeas of the larvae had specific responses to sucrose, and knockout of *HarmGR10* resulted in the larvae losing the behavioral and electrophysiological response to sucrose, indicating that HarmGR10 mediates the response of lateral sensilla styloconica to sucrose in larvae [21]. However, the GR that mediates the responses of medial sensilla styloconica to inositol in *H. armigera* larvae remains unknown.

In order to elucidate the molecular mechanisms underlying inositol detection in *H. armigera* larvae, we first characterized electrophysiological responses of the sensilla styloconica on the maxillary galea to sugars and sugar alcohols. Based on analysis of the phylogenetic tree of GRs in Lepidopteran insects, two candidate GRs for inositol detection were selected. Subsequently, we used CRISPR-Cas9 to knock out the two GR genes separately, and then examined the electrophysiological and behavioral responses of the larvae of two mutants and the wild-type to inositol. Moreover, we employed the *Xenopus* oocyte expression system and two-electrode voltage-clamping to validate necessity and adequacy of the receptor for tuning inositol. In addition, through structural modeling prediction, molecular docking, and site-specific mutagenesis, we further identified key amino acid residues for receptor-inositol interactions. All these results comprehensively reveal the cellular and molecular basis of inositol sensing in bollworm larvae.

## Results

### Electrophysiological responses of sensilla styloconica on maxillary galea of *H. armigera* larvae to sugars and sugar alcohols

**Fig 1:**
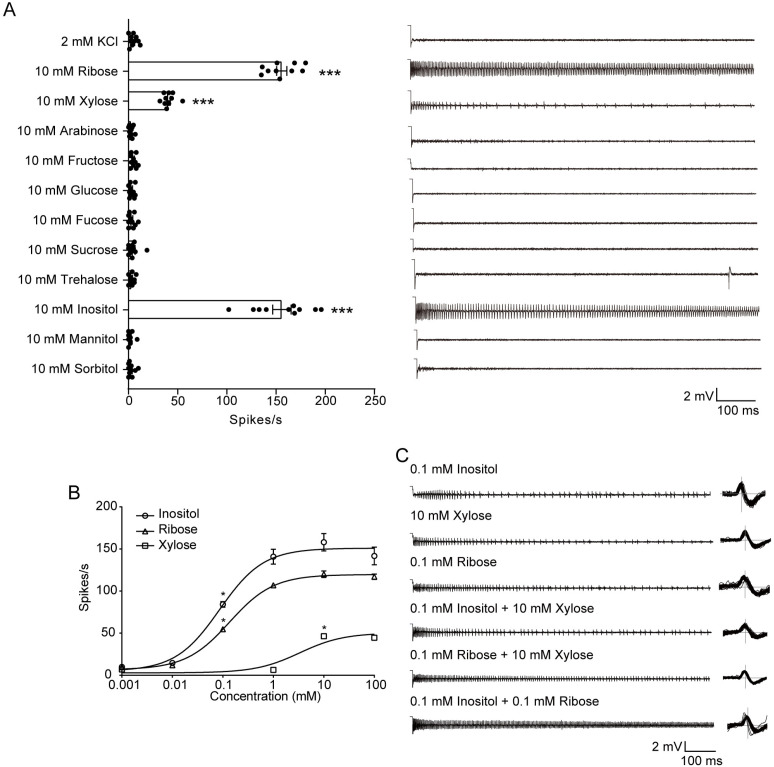
Electrophysiological responses of medial sensilla styloconica of *Helicoverpa armigera* larvae to sugars and sugar alcohols. **(A)** Left: Quantifications of firing rates of medial sensilla styloconica to representative sugars and sugar alcohols at 10 mM (n = 10; each dot represents one larva). Right: Representative spike traces of responses. Data are mean ± SEM; *p < 0.05; **p < 0.01; ***p < 0.001. Data were analyzed by independent-samples t-test (compared with control). **(B)** Dose-response curves of medial sensilla styloconica to inositol, ribose, and xylose (inositol: n = 6–9; ribose: n = 9; Xylose: n = 8). Data are mean ± SEM. Data were analyzed by one-way ANOVA with Tukey’s HSD test (p < 0.05). **(C)** Representative spike traces and waveforms of 0.1 mM inositol, 0.1 mM ribose, and 10 mM xylose, independently and binary mixtures.

We first examined the electrophysiological responses of the lateral and medial sensilla styloconica on maxillary galea in 5th instar larvae of *H. armigera* to 11 sugars and sugar alcohols, which were selected based on their abundance in plants, structural diversity, and biological relevance as primary energy sources for insects. The medial sensilla styloconica responded strongly to inositol and ribose, weakly to xylose, and had no response to other compounds (Fig 1A). Dose-response curves showed that the threshold concentrations of inositol, ribose, and xylose were 0.1 mM, 0.1 mM, and 10 mM for the medial sensilla styloconica, respectively (Fig 1B). In contrast, the lateral sensilla styloconica exhibited a strong response to sucrose and a weak response to fucose, and no responses to other compounds, consistent with the previous results (S1 Fig) [20].

To estimate whether the responses of the medial sensilla styloconica to inositol, ribose, and xylose were mediated by the same or different GSNs, we compared spiking patterns elicited by single compounds (0.1 mM inositol, 0.1 mM ribose, and 10 mM xylose) and their binary mixtures (0.1 mM inositol + 0.1 mM ribose, 0.1 mM inositol + 10 mM xylose, and 0.1 mM ribose + 10 mM xylose). Compared to the responses induced by single compounds, the mixtures induced additive responses (S2A-C Fig) with only one amplitude spikes (Fig 1C), suggesting that the same GSN in the medial sensillum styloconicum responded to inositol, ribose and xylose.

### Phylogenetic analysis

**Fig 2:**
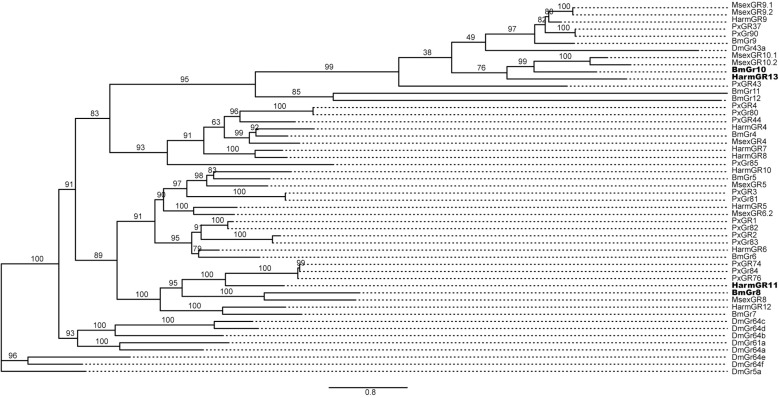
Phylogenetic tree of sugar and fructose receptors in *Drosophila* and Lepidopteran species. Dm: *Drosophila melanogaster*. Lepidoptera: Bm: *Bombyx mori*; Px: *Plutella xylostella*; Msex: *Manduca sexta*; Harm: *Helicoverpa armigera*. Receptors with bold black are inositol receptors of *B. mori* and their homologous receptors of *H. armigera.* Numbers on branches indicate ultrafast bootstrap approximation (UFBoot).

To explore the molecular basis of the medial sensilla styloconica responses to inositol, ribose, and xylose, we conducted a phylogenetic analysis of GRs from *H. armigera*, *D. melanogaster*, *B. mori*, *Plutella xylostella*, and *Manduca sexta*. The phylogenetic tree showed that HarmGR11 and HarmGR13 are orthologs of BmGR8 and BmGR10 in *B. mori* previously reported, respectively [16,17] (Fig 2). We further analyzed transcriptomic data of the maxillary galea of *H. armigera*, and the transcripts per million mapped reads (TPM) showed that only HarmGR10 exhibited the highest expression level among the sugar receptors and the DmGr43a-like receptor in the tissue, but HarmGR13 has the third highest TPM value among selected GRs and the TPM value of HarmGR11 is much lower (S3 Fig).

### Establishment of *HarmGR13* and *HarmGR11* homozygous mutants

**Fig 3:**
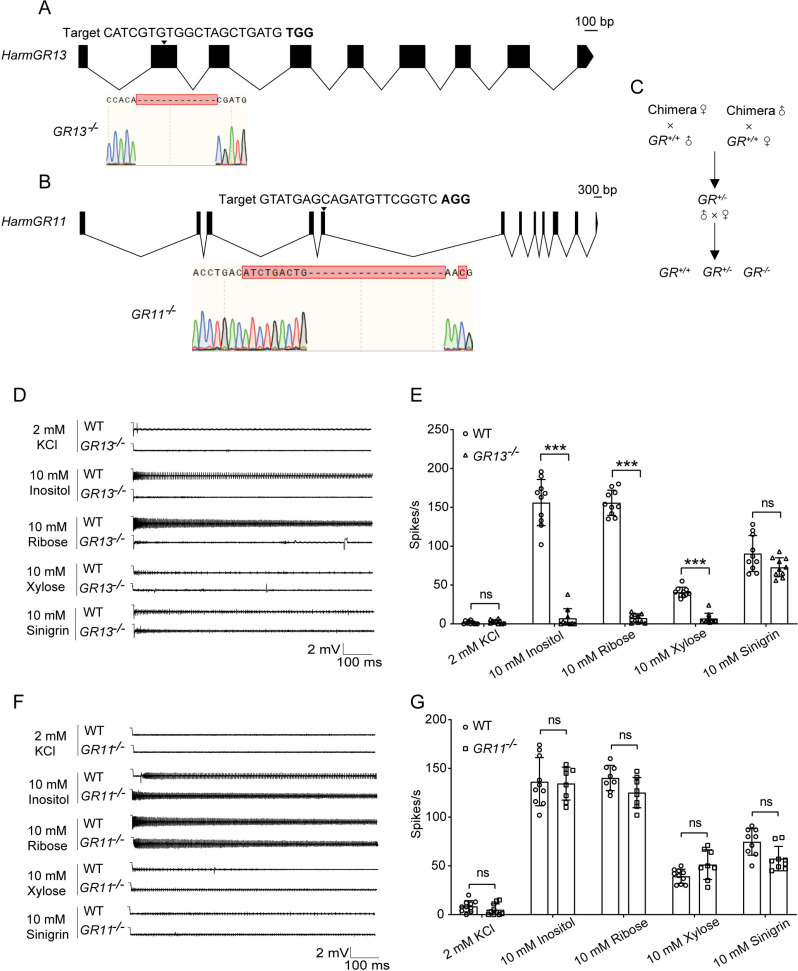
Generation of homozygous mutants for *HarmGR13* and *HarmGR11* using CRISPR-Cas9 and electrophysiological responses of medial sensilla styloconica in WT, *GR13*^*-/-*^, and *GR11*^*-/-*^ larvae to inositol, ribose, xylose, and sinigrin. **(A)** Gene structure of *HarmGR13*, target region of sgRNA, and sequencing chromatogram for homozygous mutants. **(B)** Gene structure of *HarmGR11*, target region of sgRNA, and sequencing chromatogram for homozygous mutants. **(C)** Mating process to obtain homozygous mutants. **(D)** Representative spike traces of medial sensilla styloconica of *GR13*^*-/-*^ compared to WT. **(E)** Quantifications of firing rates of WT and *GR13*^*-/-*^ to (n = 10). **(F)** Representative spike traces of medial sensilla styloconica of *GR11*^*-/-*^ compared to WT. **(G)** Response intensity of WT and *GR11*^*-/-*^ (n = 8–10). In (E) and **(G)**, each dot represents one larva; Data are mean ± SEM; *p < 0.05; **p < 0.01; ***p < 0.001. Data were analyzed by independent-samples t-test.

Using CRISPR-Cas9, we successfully generated homozygous knockout mutants for *HarmGR13* (*GR13*^*-/-*^) and *HarmGR11* (*GR11*^*-/-*^). For *GR13*^*-/-*^, a 13-bp deletion in exon 2 truncated HarmGR13 from 436 to 106 amino acids (Fig 3A). For *GR11*^*-/-*^, a 19-bp deletion and a 9-bp insertion in exon 5 resulted in the truncation of HarmGR11 from 435 to 249 amino acids (Fig 3B). Potential off-target effects were evaluated using the CRISPOR online tool. Comparison of target sequences with the *H. armigera* genome revealed no potential off-target sites with three or fewer mismatches. The two mutants showed no difference in larval weight, larval stage, pupal weight, pupal stage, and number of eggs laid compared with wild-type larvae (S4A-J Fig). It is worth noting that the larval stage of *GR13*^*-/-*^ was significantly longer than that of the wild-type (S4G Fig).

### Effects of *HarmGR13* or *HarmGR11* knockout on the electrophysiological responses of *H. armigera* larvae

We compared the electrophysiological responses of the medial sensilla styloconica on maxillary galea in larvae of *GR13*^*-/-*^, *GR11*^*-/-*^ and the wild-type to inositol, ribose, and xylose, and sinigrin at a concentration of 10 mM, and sinigrin serves as positive control. The average firing rates of *GR13*^*-/-*^ larvae to inositol, ribose and xylose were only 7.1 spikes/s, 7.4 spikes/s, 6.9 spikes/s, significantly lower than those of wild-type larvae (Fig 3D and 3E), while the responses of *GR11*^*-/-*^ larvae to the three compounds were not significantly different from those of wild-type larvae (Fig 3F and 3G). However, the responses of the two mutants to the bitter compound sinigrin were not significantly different from those of the wild-type larvae (Fig 3E and 3G). These results indicate that *HarmGR13* is involved in the taste perception of inositol, ribose and xylose by the medial sensilla styloconica of *H. armigera* larvae, while *HarmGR11* is not; None of them are involved in the taste perception of sinigrin by the medial sensilla styloconica of *H. armigera* larvae.

### Effects of *HarmGR13* or *HarmGR11* knockout on the behavioral responses of *H. armigera* larvae

**Fig 4:**
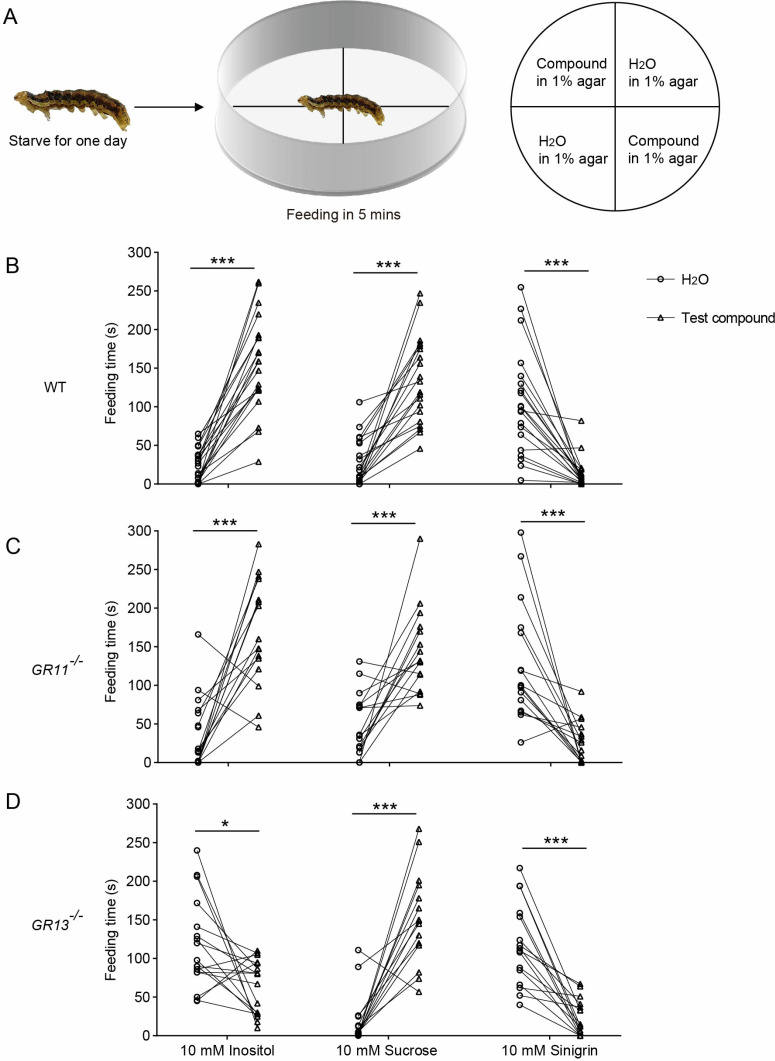
Two-choice feeding test of WT, *GR13*^*-/-*^, and *GR11*^*-/-*^ larvae. **(A)** Schematic of the two-choice feeding test. Left represents what to do with the larvae before test. Middle represents test process. Right represents the agar arrangement on petri dish. **(B)** Feeding responses of WT larvae to 10 mM inositol, sucrose, and sinigrin (n = 20–21). **(C)** Feeding responses of *GR11*^*-/-*^ larvae to the same compounds (n = 16). **(D)** Feeding responses of *GR13*^*-/-*^ larvae to the same compounds (n = 15–17). In **(B, C, **D)****: *p < 0.05; **p < 0.01; ***p < 0.001. Data were analyzed by paired t-test.

In order to further clarify the behavioral responses of *H. armigera* larvae to inositol, ribose and xylose and their relationship with HarmGR11 and HarmGR13 expression, we used two-choice tests to determine the feeding time within 5 min of the fifth-instar larvae of the wild-type, *GR11*^*-/-*^, and *GR13*^*-/-*^ on agar containing each compound and control agar (Fig 4A). Wild-type larvae fed longer on the agar containing 10 mM inositol than on the control agar, showing feeding preference of larvae for inositol (Fig 4B). However, this preference disappeared when inositol concentration was reduced to 0.01 mM (S5 Fig). The wild-type larvae had no feeding preference for ribose and xylose at 10 mM (S5 Fig). *GR11*^*-/-*^ larvae showed feeding preference for 10 mM inositol as wild-type larvae (Fig 4C). *GR13*^*-/-*^ larvae showed no feeding preference for 10 mM inositol and even a slight bias towards control agar (Fig 4D). In addition, both *GR13*^*-/-*^ and *GR11*^*-/-*^ larvae showed feeding preference for sucrose and feeding deterrence to sinigrin (Fig 4C and 4D). These results indicate that inositol has a feeding stimulating effect on *H. armigera* larvae, and HarmGR13 mediates to this behavioral response.

### Functional analysis of HarmGR13 by ectopic expression

**Fig 5:**
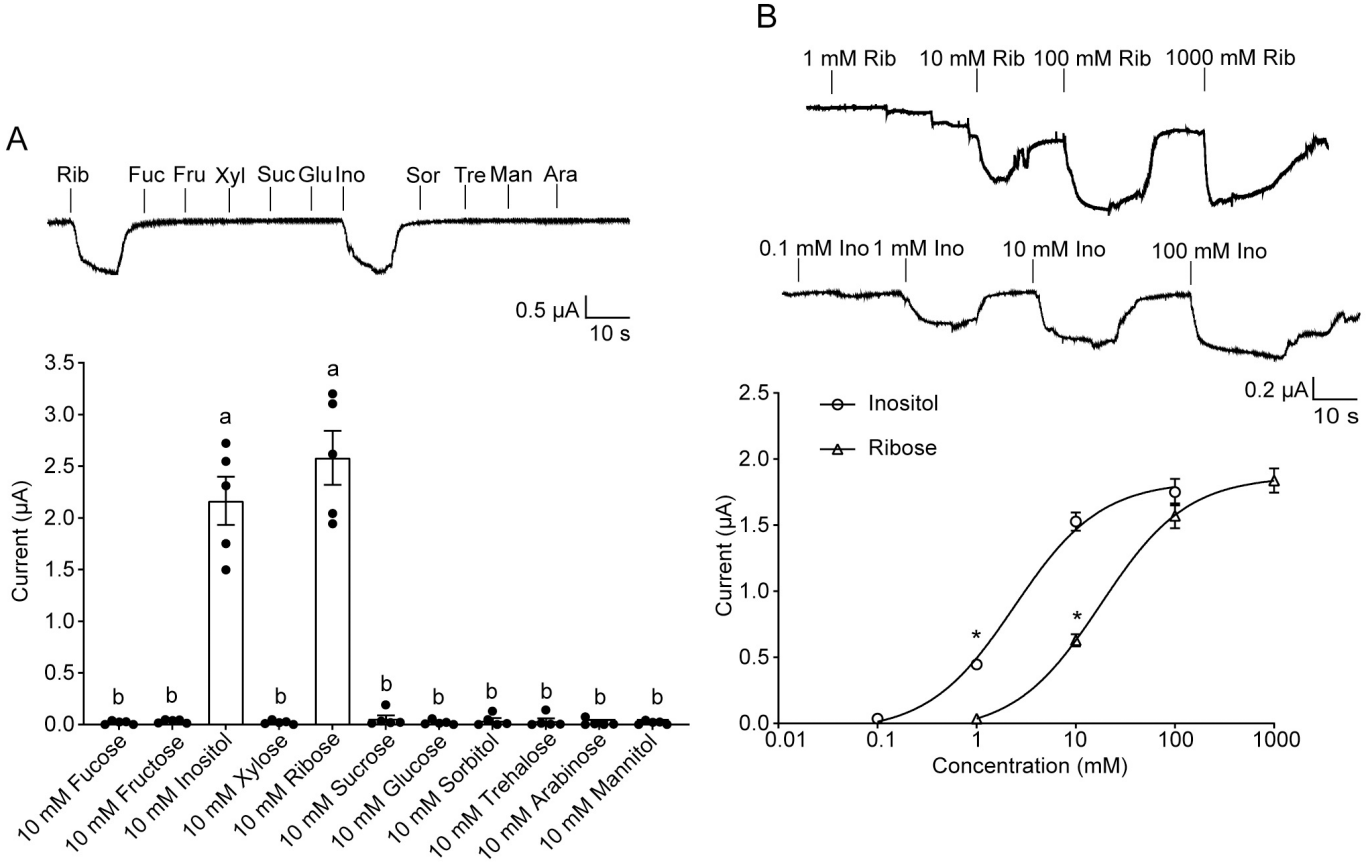
Functional characterization of HarmGR13 using the *Xenopus* oocyte two-electrode voltage-clamp system. **(A)** Top: The representative traces of HarmGR13-expressing oocytes to 11 sugars and 3 sugar alcohols at 100 mM and 10 mM concentrations. Bottom: Quantifications of the responses of GR13-expressing oocytes to each sugar and sugar alcohol (n = 5). **(B)** Top: The representative traces of HarmGR13-expressing oocytes to ribose following concentration gradient; Middle: The representative traces of HarmGR13-expressing oocytes to inositol following concentration gradient; Bottom: Dose-response curves for inositol and ribose (n = 5). In A and B Data are mean ± SEM. Data were analyzed by one-way ANOVA with Tukey’s HSD test (p < 0.05). Ara, arabinose; Fru, fructose; Fuc, fucose; Glu, glucose; Ino, inositol; Mal, maltose; Man, mannitol; Suc, sucrose; Sor, sorbitol; Tre, trehalose; Xyl, xylose.

To further validate the function of HarmGR13 ectopically, we reverse-transcribed the gene into cRNA and injected it into *Xenopus* oocytes. After incubating the oocytes at 18°C for two days, inward currents elicited by stimulation with 11 sugars and sugar alcohols were recorded by using two-electrode voltage-clamp. The results demonstrated that the oocytes expressing HarmGR13 showed strong responses to 10 mM inositol and 100 mM ribose (Fig 5A), with inward current amplitudes of 1.98 ± 0.6 μA and 2.58 ± 0.52 μA, respectively (Fig 5A). The dose-response curves showed that the threshold concentrations of inositol and ribose evoked responses were 1 mM and 10 mM, respectively (Fig 5B).

**Fig 6:**
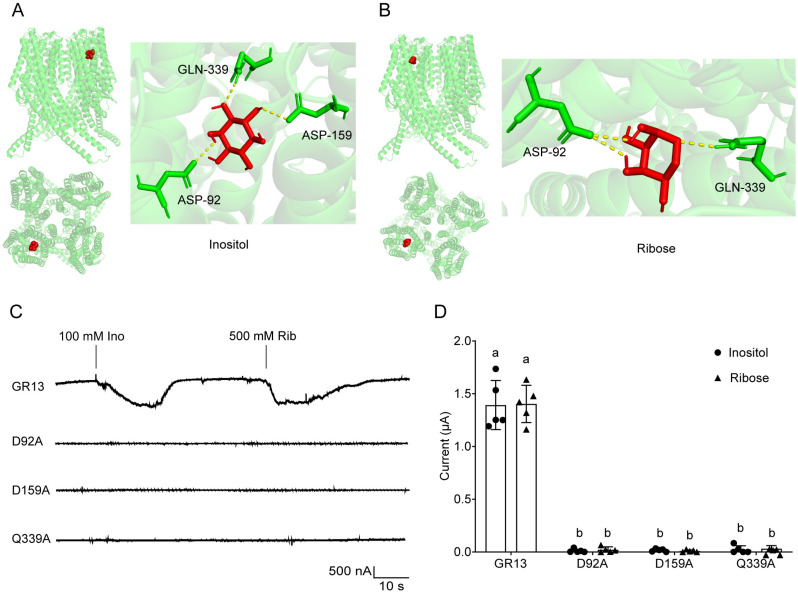
Predicted HarmGR13 structure and ligand-binding analysis through site-directed mutagenesis. **(A, B)** Predicted 3D protein structure of HarmGR13 and molecular docking with inositol and ribose. **(C)** Representative traces of oocytes expressing GR13 and its point mutants (D92A, D159A, and Q339A) to 100 mM inositol and 500 mM ribose. **(D)** Quantifications of the responses of oocytes expressing HarmGR13 and its mutants to 100 mM inositol and 500 mM ribose (n = 5). Data are mean ± SEM and were analyzed by one-way ANOVA with Tukey’s HSD test (p < 0.05).

According to the phylogenetic tree of GRs, HarmGR13 belongs to the same clade as BmGr9 from *B. mori*, MsexGR10.1 from *M. sexta,* and DmGr43a from *D. melanogaster* (Fig 2). Based on the homotetrameric structure of BmGr9 recently resolved [22], we predicted the structure of HarmGR13 using AlphaFold3. The resulting model had an ipTM score of 0.78 and a pTM score of 0.8, indicating the predicted structure is highly reliable. Using the AutoDock Vina algorithm, we further performed molecular docking simulations of the structure with the two ligands, and the estimated binding energies of HarmGR13 to inositol and ribose are -6.2 kcal/mol and -5.2 kcal/mol, respectively. Polar interaction analysis indicated that HarmGR13 forms polar contacts with inositol at Asp92, Asp159, and Gln339, and with ribose at Asp92 and Gln339 (Fig 6A and 6B). These residues are conserved across HarmGR13, BmGR10, and MsexGR10.1 (S6 Fig).

To test the functional significance of Asp92, Asp159, and Gln339 in HarmGR13, we performed site-specific mutagenesis at each site and substituted them with the non-polar amino acid alanine. The unmutated *HarmGR13* and three single-site mutated genes were transcribed into cRNA and injected into *Xenopus* oocytes for functional analysis. The oocytes expressing the unmutated HarmGR13 exhibited inward currents in response to 100 mM inositol and 500 mM ribose, but the oocytes expressing each of three mutated HarmGR13 did not show any inward currents in response to the same stimuli (Fig 6C and 6D), indicating that Asp92, Asp159, and Gln339 are essential for HarmGR13 binding to inositol and ribose.

## Discussion

In this study, we demonstrate that HarmGR13 functions as a receptor tuned to inositol and ribose in the medial sensilla styloconica of *H. armigera* larvae. Additionally, we identified three amino acid residues critical for HarmGR13 binding to inositol as a feeding stimulant for larvae of *H. armigera*. These results may have general implications for understanding why larvae of many lepidoptera species have the ability to detect myo-inositol.

Myo-inositol is a ubiquitous metabolite of plants. It plays a crucial role in physiological processes of plant growth and development, including phosphate storage, cell wall biosynthesis, production of stress-related molecules, intercellular communication, and storage and transport of plant hormones. With a sweetness similar to sugar, inositol is an indispensable nutrient and energy source and feeding stimulus for herbivorous insects [23].

Lepidoptera is an important model for studying the taste perception of inositol because caterpillars of many species are sensitive to inositol [24]. Behavioral studies have shown that myo-inositol induces feeding and extends feeding duration in *B. mori* larvae [25,26]. Similarly, myo-inositol has been observed to stimulate feeding and suppress bitter taste perception in *M. sexta* larvae [27,28]. Most of the electrophysiological studies focused on two styloconic sensilla on the maxillary galea, which play a critical role in host plant selection of caterpillars. In *B. mori*, *Operophtera brumata*, and *Adoxophyes orana*, the lateral sensilla styloconica are sensitive to inositol. In *Heliothis virescens*, *H. armigera*, *Helicoverpa assulta*, *Mamestra configurata*, *Mamestra brassicae*, S*podoptera exempta*, and *Spodoptera litura*, the medial sensilla styloconica are sensitive to inositol. In *M. sexta*, *Euproctis phaeorrhoea*, and *Philosamia cynthia*, both the medial and lateral sensilla styloconica are sensitive to inositol [14].

Given the general sensitivity of caterpillars to inositol, elucidating the molecular mechanisms underlying taste perception of inositol is pivotal for understanding host-plant recognition in Lepidopteran insects. However, until now the GRs responsible for inositol detection in caterpillars remains unclear. There are only three related studies on *B. mori*. Zhang et al. (2011) [16] reported that BmGr8 functions independently in Sf9 cells and responds in a concentration-dependent manner to myo-inositol and epi-inositol, which is the first chemoreceptor shown to respond specifically to inositol. Employing *Xenopus* oocyte expression system with two-electrode voltage-clamp and HEK293T cell expression systems with the fluorescent Ca2^+^ indicator GCaMP3, Kikuta et al. (2016) [17] found that the cells expressing BmGr10 specifically respond to myo-inositol and epi-inositol, but the cells expressing BmGR8 did not. It is worth noting that the latter study used a BmGR8 variant lacking 38 amino acids compared to the former study. Recently, it has been further proved that BmGr10 is a putative inositol receptor in the epipharyngeal sensillum using HEK293T cells with the fluorescent Ca2^+^ indicator Flur-4 [29]. Endo et al. (2024) [30] used the HEK293T cell expression system and found that BmGR9 and BmGR6 were highly sensitive to mulberry compounds such as chlorogenic acid and isoquercetin, and also to inositol at concentrations as low as 10 pM and 1 nM, respectively. However, inositol might not be the primary ligand for these receptors, as the threshold concentrations for inositol differed significantly from those for other ligands. In the above studies, the function of related GRs was not verified *in vivo*.

Gene editing techniques have been widely used in model systems such as *Drosophila* for *in vivo* analysis of gene function, but their application in Lepidoptera insects remains limited. CRISPR-Cas9 can completely eliminate the expression of a given GR gene in insects, and this is usually not lethal to insects, so it is a highly effective tool to verify the function of a certain GR *in vivo*. In this study, we used CRISPR-Cas9 to knock out *HarmGR11* and *HarmGR13* in *H. armigera* larvae to verify the function of these two genes *in vivo*. We found that knockout of *HarmGR13* resulted in loss of the electrophysiological responses to inositol, ribose and xylose in the medial sensilla styloconica of the larvae, and the larvae no longer had a feeding preference for inositol, while knockout of *HarmGR11* did not cause any changes in the electrophysiology and behavior of the larvae, indicating that HarmGR13 is indispensable for inositol detection. Unexpectedly, in the behavior test, *GR13*^*-/-*^ larvae even showed a slight bias to control agar, which might be caused by the compensatory changes in peripheral taste after the knockout of *HarmGR13*. Furthermore, *GR13*^*-/-*^ larvae grows much slower than WT larvae. This is very likely due to the loss of inositol perception ability, which affects the feeding efficiency and nutrient intake of larvae. Using the *Xenopus* oocyte expression system and two-electrode voltage clamp, we further reveal that expression of HarmGR13 alone is sufficient for the cell responding to inositol and ribose. However, the function of HarmGR11 remains unclear. A previous study indicated that BmGr8, the homolog of HarmGR11, was highly expressed in the testis of *B. mori* [16]. In this study, we find that HarmGR11 is not functional in the medial sensilla styloconica of *H. armigera* larvae. Moreover, knocking out *HarmGR11* has no effect on the reproductive capacity of *H. armigera* adults.

There are only two contact chemosensilla on the maxillary galea of *H. armigera* larvae: the lateral sensillum styloconicum is sensitive to sucrose, and the medial sensillum styloconicaum is sensitive to inositol. Our recent work found that HarmGR10 mediates sucrose sensing in the lateral sensillum styloconicum, and this study reveals that HarmGR13 mediates inositol sensing in the medial sensillum styloconicum. The threshold concentrations of evoking responses of the two sensilla were all 0.1 mM, but the expression level of HarmGR10 is significantly higher than that of HarmGR13. This can be explained by the fact that HarmGR10 is less sensitive to sucrose than HarmGR13 to inositol. Ectopic expression experiments showed that the threshold concentration of sucrose for HarmGR10 was 100 mM, while that of inositol for HarmGR13 was 1 mM. Therefore, the response intensity of a given GRN to a ligand mainly depends on the expression level of the GR, the binding affinity of the GR to the ligand, and the concentration of the ligand.

The results of our behavioral, electrophysiological and molecular experiments are well matched for inositol, but less so for ribose and xylose. This discrepancy may be due to the different sensitivities of the inositol GSN to the three compounds, or the involvement of other GSNs sensitive to ribose or xylose. Ribose is a vital component of nucleic acids and participates in the pentose phosphate pathway and ATP synthesis, serving as both a substrate for energy production (ATP) and a signaling molecule in bacterial quorum sensing [31]. Studies on insect ribose perception are limited. In *Drosophila* larvae, ribose attracts feeding behavior and is detected by the Gr28 cluster genes, while in adults, ribose induces aversion, mediated by GSNs in s-type sensilla and bitter receptors [32,33]. Research on xylose perception in insects is also scarce. In nature, xylose is a major component of plant cell walls and a primary sugar in some plant nectars [34,35]. However, pollinating insects, such as Cape honeybees (*Apis mellifera capensis*), exhibit aversion to xylose [36]. The mechanism of sensing ribose and xylose in *H. armigera* larvae needs further study.

We used AlphaFold3 to predict the HarmGR13 structure in reference to the recently resolved structure of the fructose receptor BmGR9 in *B. mori* [22]. Since HarmGR13 and BmGR9 belong to the same subfamily of DmGr43a homologues, the predicted structure is expected to be relatively accurate. Gaining insight into how HarmGR13 interacts with ligands such as inositol, we confirmed three key amino acid residues involved in ligand binding by site-directed mutagenesis. However, during molecular docking, the predicted docking amino acid residues may not be exactly consistent with actual binding interactions. Although the mutation of the predicted residue led to the loss of HarmGR13 function, other residues may also be involved in the binding process. Understanding in detail how these residues interact with ligands and open ion channels requires resolving the actual three-dimensional structure of the receptor using the cryo-electron microscopy.

In this work, by using behavior assays, electrophysiology, CRISPR-Cas9, ecotopic expression, molecular docking, and site-specific mutagenesis, we convincingly reveal that HarmGR13 is indispensable and sufficient for tasting inositol and ribose in the medial sensilla styloconica of *H. armigera* larvae and three amino acid residues are critical for HarmGR13 functioning, and confirm that inositol has a stimulating effect on the feeding of larvae. Understanding the cellular and molecular mechanisms of inositol detection in *H. armigera* larvae is of great significance for further understanding host-plant selection of this serious crop pest and other Lepidoptera insects. It also offers new pathways for designing effective and environmentally friendly control strategies. In addition to larvae, the F5a sensilla in the foreleg tarsus of *H. armigera* adults also have electrophysiological responses to inositol, which can trigger proboscis extension reflex and prolongs proboscis extension duration [37]. Whether inositol detection in *H. armigera* adults is also mediated by HarmGR13 needs further study.

## Materials and methods

### Ethics statement

All experiments were approved by the Animal Care and Use Committee of the Institute of Zoology, Chinese Academy of Sciences, in accordance with laboratory animal care guidelines (IOZ17090-A).

### Insects and *Xenopus laevis*

*H. armigera* used in the study is a laboratory-maintained wild-type strain reared under controlled conditions (28 ± 1 °C, 30%-50% relative humidity). Larvae are fed an artificial diet (nutrients include wheat bran, yeast, and tomato paste) in glass tubes. Once the larvae reach the third instar, they are transferred to a 24-well plate for individual rearing. After pupation, they are placed in a cage covered with gauze until they reach sexual maturity, at which point mating occurs, and eggs are laid on the gauze. The egg gauze is then collected and disinfected with 10% formaldehyde for 5 minutes before being dried and stored for future larval hatching and rearing. *Xenopus laevis* is reared in the indoor breeding facility at Shanxi University, with a temperature of 20 ± 1°C and approximately 50% humidity. They are fed fish feed, with water changes every two days and feeding every four days.

### Chemicals

Total 13 chemicals, including representative sugar and sugar alcohols (pentose, hexose, disaccharide and sugar alcohol), sinigrin and potassium chloride were used in tip-recoding and two-electrode voltage-clamping. Each chemical was of the highest purity available commercially. The detailed information of each compound was compiled in S1 Table.

### Tip-recording

The tip-recording technique is used to record the taste sensilla responses of *H. armigera* larvae based on previously published methods [38,39]. The test compounds are introduced into a glass capillary in contact with the sensilla, with a silver wire inserted as a recording electrode. The capillary opening has a diameter of approximately 50 μm, encompassing the entire sensillum. Action potentials (spikes) generated within the first second of stimulation are amplified by a preamplifier and sampled using a computer equipped with a Metrabyte DAS16 A/D conversion board. The first stage of amplification utilizes an AD 515-K (Analog Devices) integrated circuit, producing an input bias current of <1 pA, with an input impedance of 1015 ohms and 0.8 pF. A GO-box interface is used for signal conditioning, incorporating a second-order band-pass filter (-3 dB frequencies: 180 and 1700 Hz). All tested compounds are dissolved in 2 mM potassium chloride, with 2 mM potassium chloride serving as a negative control. All solution concentrations for testing reactions are set at 10 mM. Sugars that elicit significant electrophysiological responses from the taste sensilla are tested in a dose-response from low to high concentrations (0.01 mM, 0.1 mM, 1 mM, 10 mM, and 100 mM). To prevent adaptation of the sensilla to the stimulating compounds, the time interval between different compound stimulations on the same sensillum is at least three minutes. Digital signals are stored using SAPID 16.0 software, and the data is analyzed with AutoSpike 3.7 software (Syntech, Buchenbach, Germany). Spikes are classified based on typical biphasic waveforms and peak amplitudes, with frequency counts of the same type of spikes starting from the first second after stimulation. electrophysiological recordings from the larvae are conducted using first-day fifth-instar larvae. To ensure that all experimental larvae are at the same developmental stage, fourth-instar larvae near molting are selected and transferred to glass tubes, where they are fed green peppers. Prior to electrophysiological recordings, the test larvae are starved for approximately two hours. The head of the test insect is cut and fixed onto a circular silver wire support, which is connected to the amplifier via a copper microconnector. The sensilla styloconica on the maxillary of the larvae are exposed to assess electrophysiological responses.

### Phylogenetic analysis of sugar GRs

The amino acid sequences of fructose and sugar receptors annotated of *H.armigera* and those annotated by *Drosophila melanogaster*, *B. mori*, *P. xylostella* and *M. sexta* were used to construct phylogenetic trees [40–43]. MAFFT v7.455 [44] was used to make sequence alignment of amino acid sequences. Phylogenetic trees were reconstructed using a maximum-likelihood method implemented in IQ-TREE v2.2.0 [45] under the Jones-Taylor-Thornton (JTT)+F + G4 model for 5000 ultrafast bootstraps. FigTree v1.4.4 was used to visualize and edit the constructed phylogenetic tree.

### *In vitro* synthesis of single-guide RNA

Target sites for sgRNA of *HarmGR11* and *HarmGR13* were selected using CRISPOR online website (http://crispor.tefor.net/). For *HarmGR11*, the sgRNA target site was in the fifth exon, and for *HarmGR13*, it was in the second exon. The design principles of sgRNA primers are detailed in S2 Table, according to the requirements of the GeneArt Precision gRNA Synthesis Kit (Invitrogen, Vilnius, Lithuania), and synthesized following the GeneArt gRNA Prep Kit (Invitrogen, Vilnius, Lithuania) protocol. Purification was carried out with the GeneArt gRNA Clean Up kit (Invitrogen, Vilnius, Lithuania), and the final sgRNA products were dissolved in RNase-free water and stored at -80 °C until use.

### Embryo microinjection

The synthesized sgRNA was diluted to 200 ng/μL, and TrueCut Cas9 protein (Invitrogen, Vilnius, Lithuania) was diluted to 500 ng/μL sgRNA and Cas9 protein were mixed at a 4:1 ratio, resulting in final concentrations of 180 ng/μL sgRNA and 100 ng/μL Cas9 protein. Freshly fertilized *H. armigera* eggs (within 1hour post-laying) were aligned on a slide with double-sided tape. The sgRNA-Cas9 mix was injected into prepared eggs using a PLI-100A PICO-INJECTOR (Warner Instruments, Hamden, Connecticut, USA). Injected eggs were placed in a 10 cm Petri dish with moist filter paper, maintained at 26 °C for larval hatching, and hatched larvae were individually reared in glass tubes with artificial diet.

### DNA extraction and mutagenesis detection

To screen for homozygous mutants of *HarmGR13* and *HarmGR11*, the tarsus of the hind legs from adults on their first day post-emergence was used to extract genomic DNA for sequence amplification of sgRNA target sites. Genomic DNA was extracted using the TIANcombi DNA Lyse&Det PCR Kit (TIANGEN Biotech, Beijing, China). For PCR, a 25 μL reaction system was prepared with 1.5 μL DNA template, 1 μL of each primer, 12.5 μL of 2 × Rapid Taq Master Mix (Vazyme, China), and 9 μL of ddH2O. The PCR program was 95 °C for 3 min (initial denaturation), followed by 35 cycles (95 °C for 15 s, 57 °C for 30 s for *HarmGR11* or 55 °C for 15 s for *HarmGR13*, and 72 °C for 10 s), and a final extension at 72 °C for 5 min. PCR products were sent to SinoGenoMax for Sanger sequencing, and results were analyzed with SnapGene software (from Insightful Science; available at snapgene.com). Primier for mutagenesis detection is in S2 Table.

### Screening homozygous mutants

Homozygous mutant lines *GR11*^*-/-*^ and *GR13*^*-/-*^ were established respectively. To obtain *GR11*^*-/-*^, G0 chimeras were backcrossed with wild-type individuals to produce G1 offspring; heterozygous G1 individuals with a 19 bp deletion and 9 bp insertion on one chromosome were then self-crossed to yield homozygous *HarmGR11* mutants in the G2 generation, which were self-crossed to generate G3 homozygotes. The same protocol was used for *GR13*^*-/-*^, with a 13 bp deletion on one chromosome in the G1 heterozygotes. Mutation rates for each generation are provided in the S3 Table.

### Off-target effects detection

To verify the absence of off-target effects in homozygous mutants *GR11*^*-/-*^ and *GR13*^*-/-*^, potential off-target sites were predicted as described using CRISPOR (http://crispor.tefor.net/) to match sgRNA targeting sites with the *H. armigera* genome [40]. Results showed no potential off-target sites with less than three mismatches. Further validation followed published methods [21,46] included comparing developmental time, weight, pupal weight and duration, and oviposition rates between wild-type and mutant strains. Randomly selected wild-type, *GR11*^*-/-*^ and *GR13*^*-/-*^ larvae were tested. Developmental time was recorded from hatching to the fifth instar and to pupation; larval weight was measured on the first day of the fifth instar, pupal weight on 3–4 days after pupation, and the number of eggs laid by a female when paired with two males was determined.

### Effects of *HarmGR13* or *HarmGR11* knockout on contact chemoreception in larvae

For wild-type, *GR11*^*-/-*^ and *GR13*^*-/-*^ larvae, tip recording methodology was the same as describe above and slightly adapted for testing compounds (e.g., inositol, ribose, xylose, and sinigrin). Sinigrin triggered responses in the bitter GRN of the medial sensilla styloconica [46] and served as a positive control, while 2 mM potassium chloride served as a negative control for all sensilla.

### Effects of *HarmGR13* or *HarmGR13* knockout on feeding behaviors of larvae

We adapted a previously reported two-choice assay [21]. Fourth-instar *H. armigera* larvae at the pre-molting stage were transferred to fresh glass tubes and starved overnight to ensure they remained unfed until the experiment. Inositol, ribose, and xylose were dissolved to the required concentrations and mixed with 30 ML of 1% agar, while water agar served as the control. Two opposing sectors of compound agar and water agar were placed in a 10 cm Petri dish, filling the dish entirely. Wild-type and mutant larvae were introduced to the dish and allowed to feed. The duration of continuous feeding on each agar block was recorded over 5 minutes. Initial test concentrations were set at 10 mM, with concentration gradient experiments conducted at 0.01 mM, 0.1 mM, 1 mM, and 10 mM.

### RNA extraction and cDNA synthesis

The galea of larval of *H. armigera* were dissected and placed in 1 mL of QIAzol Lysis Reagent (QIAGEN, Hilden, Germany). Total RNA was extracted following the kit’s protocol. The quality and concentration of RNA were measured using a NanoDrop 2000 spectrophotometer (Thermo Fisher Scientific, Waltham, USA). For cDNA synthesis, 0.1 μg of total RNA was used, following the steps in the reverse transcription kit (Thermo Fisher Scientific, Waltham, USA). The cDNA was dissolved in ddH2O and stored at -20 °C for further use.

### Gene cloning of sugar GRs

Specific primers were designed based on transcriptome data from maxillary galea of *H. armigera* larval and previously reported sugar receptor gene sequences in the NCBI database [21,47,48]. GenBank accession numbers and specific primers for cloning full-length sequences are provided in S2 Table. Gene cloning was performed with Q5 High-Fidelity DNA Polymerase (New England Biolabs, Beverly, MA, USA) in a 25 μL PCR reaction system: 5 μL 5 × Q5 Reaction Buffer, 0.5 μL 10 μM dNTP, 1.25 μL of each primer, 15.75 μL H2O, 1 μL cDNA, and 0.25 μL Q5 High-Fidelity DNA polymerase. The PCR program was 98 °C for 10 s, 35 cycles (98 °C for 10 s, 60 °C for 30 s, 72 °C for 1 min), with a final extension at 72°C for 2 min. PCR products were separated by agarose gel electrophoresis, and target bands were excised and recovered using a gel extraction kit (TransGen Biotech, Beijing, China). The extracted products were ligated into the pEASY-Blunt3 vector (TransGen Biotech, Beijing, China), propagated in E. coli, and plasmids were sequenced at SinoGenoMax for Sanger sequencing. Sequence integrity and similarity to the reported sequence were confirmed, and no alternative splicing forms were detected during sequence analysis or cloning.

### Ectopic expression and functional analysis of GRs

Full-length sugar receptor sequences were PCR-amplified, introducing restriction enzyme sites and a Kozak sequence, and cloned into the pGEM-T vector. After digestion with suitable enzymes, sequences were subcloned into the pCS2 + vector, linearized, and cRNA synthesized using the mMESSAGE mMACHINE SP6 kit (Ambion, Austin, TX, USA), diluted to 2000 ng/μL in ddH2O, and stored at -80 °C. *Xenopus* oocytes dissection followed established methods [49], with anesthetized frogs on crushed ice. Oocytes were isolated and treated with 2 mg/mL collagenase type I (Sigma-Aldrich, St Louis, MO, USA) for 30 min at room temperature. HarmGR13 was expressed in oocytes via cRNA injection using a NANOJECT III (Drummond Scientific Company, Broomall, PA, USA) with a total injection volume of 27.6 nL (approx. 80 ng at 10 nL/s). Negative controls included oocytes injected with ddH2O. Oocytes were incubated for 2 days at 18 °C in Ringer’s solution (96 mM NaCl, 2 mM KCl, 1 mM MgCl2, 1.8 mM CaCl2, 5 mM HEPES, pH 7.6). Whole-cell currents were recorded using a two-electrode voltage clamp, with glass electrodes filled with 3 M KCl and resistance set at 0.2-2.0 MΩ. Signals were amplified with an OC-725C amplifier (Warner Instruments,Hamden, CT, USA) and maintained at -80 mV, low-pass filtered at 50 Hz and digitized at 1kHz. A total of 11 representative sugars and sugar alcohols were tested. All compounds were dissolved in 1 × Ringer solution at 100 mM or 10 mM as the initial test concentration. The compounds were made to stimulate clawed frog oocytes by perfusion for about 20 s per stimulation in random order and 1 × Ringer rinses. Dose-response tests were performed for inositol and ribose in order of low to high concentration (0.01 mM, 0.1 mM, 1 mM, 10 mM, 100 mM, 1000 mM, with myo-inositol supersaturating at a concentration of 1000 mM). Data were acquired and analyzed using the instrument Digidata 1322A and the software pCLAMP 10.4.2.0 (Axon Instruments Inc., Foster City, CA, USA).

### Molecular docking

We utilized AlphaFold3 [50] to predict the 3D structure of the HarmGR13. HarmGR13 was modeled using its amino acid sequence. The 3D structures of ligand compounds were downloaded from the PubChem database (https://pubchem.ncbi.nlm.nih.gov/) and converted into docking-compatible formats using Open Babel version 3.1.1 [51] and AutoDock [52]. Next, the predicted receptor 3D structures were processed using PyMOL version 3.1 (The PyMOL Molecular Graphics System, Version 3.1 Schrödinger, LLC.) and AutoDock. For molecular docking, the receptor and ligands were imported into AutoDock, and the docking regions were defined. Docking was performed using the AutoDock Vina algorithm [53,54]. Docking visualizations, graphical editing and indentification of polar-interaction amino acid residues were conducted in PyMOL version 3.1.

### Site-directed mutagenesis

Site-directed mutagenesis was performed on amino acid residues identified through molecular docking analysis. Primers were designed to cover the mutation site, with the codon at the target amino acid position changed to GCC (alanine codon) and approximately 20 bp overlaps. Using 2 × Phanta Max Master Mix (Vazyme, China) and high-fidelity polymerase, plasmids were linearized via PCR, the primer sequence is provided in S2 Table. The amplification program consisted of the following steps: 95 °C for 3 minutes, 35 cycles of 98 °C for 15 seconds, 60 °C for 15 seconds, and 72 °C for 3 minutes, followed by a final extension at 72 °C for 5 minutes. The PCR products were purified and transformed into competent cells, and single bacterial colonies were screened to obtain site-directed mutagenesis plasmids. The plasmids were sent to Beijing Tsingke Biotech Co., Ltd. for Fast NGS sequencing to confirm plasmid integrity and the single amino acid mutation. The mutagenesis plasmids were then ectopically expressed in *Xenopus* oocytes, and inward currents of inositol and ribose under perfusion were recorded.

### Data analysis and graph generation

Statistical analyses were performed using GraphPad Prism 9.0.0 (Dotmatics, San Diego, CA, USA). For tip recordings, data comparisons between test compounds and controls were conducted using independent-samples t-tests. Data from two-choice assays were analyzed using paired-samples t-tests. Concentration gradient recordings and two-electrode voltage-clamp data were analyzed via one-way ANOVA followed by Tukey’s HSD test for multiple comparisons. All graphs were generated using GraphPad Prism 9.0.0, with subsequent graph layout made in Adobe Illustrator 2020 (Adobe Systems, San Jose, CA, USA).

## Supporting information

S1 Fig Electrophysiological responses of lateral sensilla styloconica to sugars, sugar alcohols. (A) Left: Quantifications of firing rates of lateral sensilla styloconica to representative sugars and sugar alcohols at 10 mM (n = 10; each dot represents one larva). Right: Representative spike traces of responses. Data are presented as mean ± SEM; *p < 0.05; **p < 0.01; ***p < 0.001. Data were analyzed by independent-samples t-test (compared with control).
(TIF)

S2 Fig Quantifications of firing rates of medial sensilla styloconica for mixtures of 0.1 mM inositol, 0.1 mM ribose, and 10 mM xylose. (A-C) Quantifications of firing rates of medial sensilla styloconica for 0.1 mM inositol, 0.1 mM ribose, and 10 mM xylose and their binary mixture (n = 9). Data are mean ± SEM. Data were analyzed by one-way ANOVA with Tukey’s HSD test (p < 0.05).
(TIF)

S3 Fig Expression patterns of taste receptors in maxillary galea of larvae of *Helicoverpa armigera.* TPM values of 10 annotated sugar and fructose receptors obtained via transcriptome sequencing (n = 3). Data are mean ± SEM and analyzed by one-way ANOVA with Tukey’s HSD test (p < 0.05).
(TIF)

S4 Fig Off-target effect assessment in *GR13*^*-/-*^ and *GR11*^*-/-*^. (A-E) Comparisons between *GR13*^*-/-*^ and WT in larval weight, larval stage, pupal weight, pupal stage, and number of eggs laid. (F-J) Comparisons between *GR11*^*-/-*^ and WT for the same parameters. A, C, D n = 17; B n = 27,14; E, J n = 5; G, I n = 15; G n = 12; H n = 20; (A–J) Data are mean ± SEM; ns, p > 0.05; *p < 0.05; **p < 0.01; ***p < 0.001. Data were analyzed by independent-samples t-tests.
(TIF)

S5 Fig Additional two-choice feeding test in WT larvae. Feeding responses to inositol at 1 mM, 0.1 mM, and 0.01 mM (n = 19–21); ribose at 10 mM (n = 24); and xylose at 10 mM (n = 23). Each line represents one larva. *p < 0.05; **p < 0.01; ***p < 0.001. Data were analyzed by paired t-test.
(TIF)

S6 Fig Amino acid sequence alignment of three homologous receptors: BmGR10, HarmGR13, and MsexGR10.1. Arrows indicate key residues identified via molecular docking: D92, D159, and Q339. Red arrows denote residues involved in polar interactions with both inositol and ribose; green arrows denote residues interacting only with inositol.
(TIF)

S1 Table Chemicals used in the experiment. (XLSX)

S2 Table Primer sequences used for the experiment. (XLSX)

S3 Table Summary of the CRISPR-Cas9 mutation rates. (XLSX)

S4 Table Raw data used in the figures and statistical analyses. (XLSX)
