# Supplementary figures and images for: HarmGR13 mediates myo-inositol taste perception in Helicoverpa armigera larvae
Source: PLoS Genet. 2025 Jun 3;21(6):e1011744. doi: 10.1371/journal.pgen.1011744 (PMC12165416; doi:10.1371/journal.pgen.1011744)

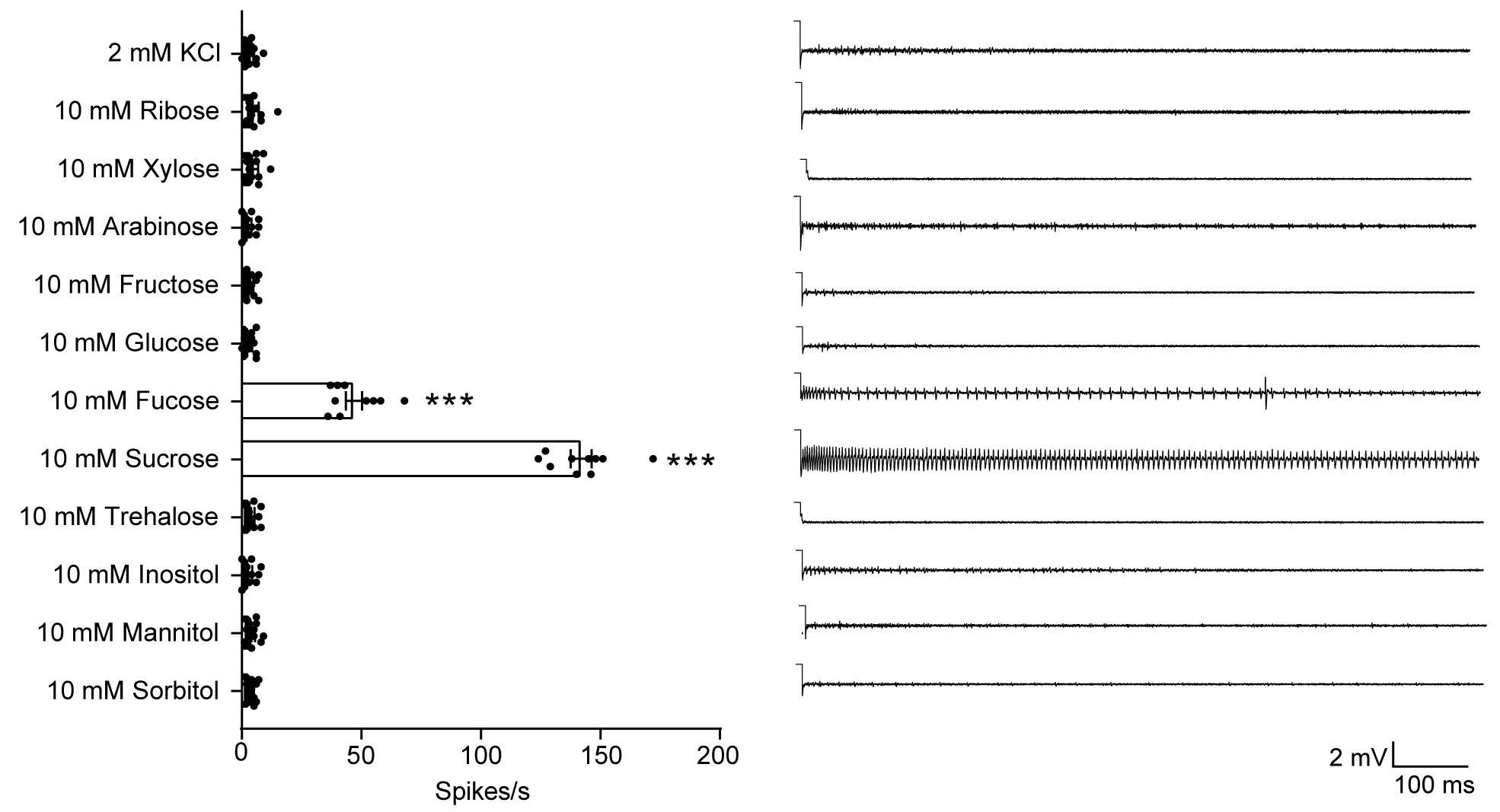

Supplement: S1 Fig — (A) Left: Quantifications of firing rates of lateral sensilla styloconica to representative sugars and sugar alcohols at 10 mM (n = 10; each dot represents one larva). Right: Representative spike traces of responses. Data are presented as mean ± SEM; *p < 0.05; **p < 0.01; ***p < 0.001. Data were analyzed by independent-samples t-test (compared with control). (TIF) [file pgen.1011744.s001.tif]

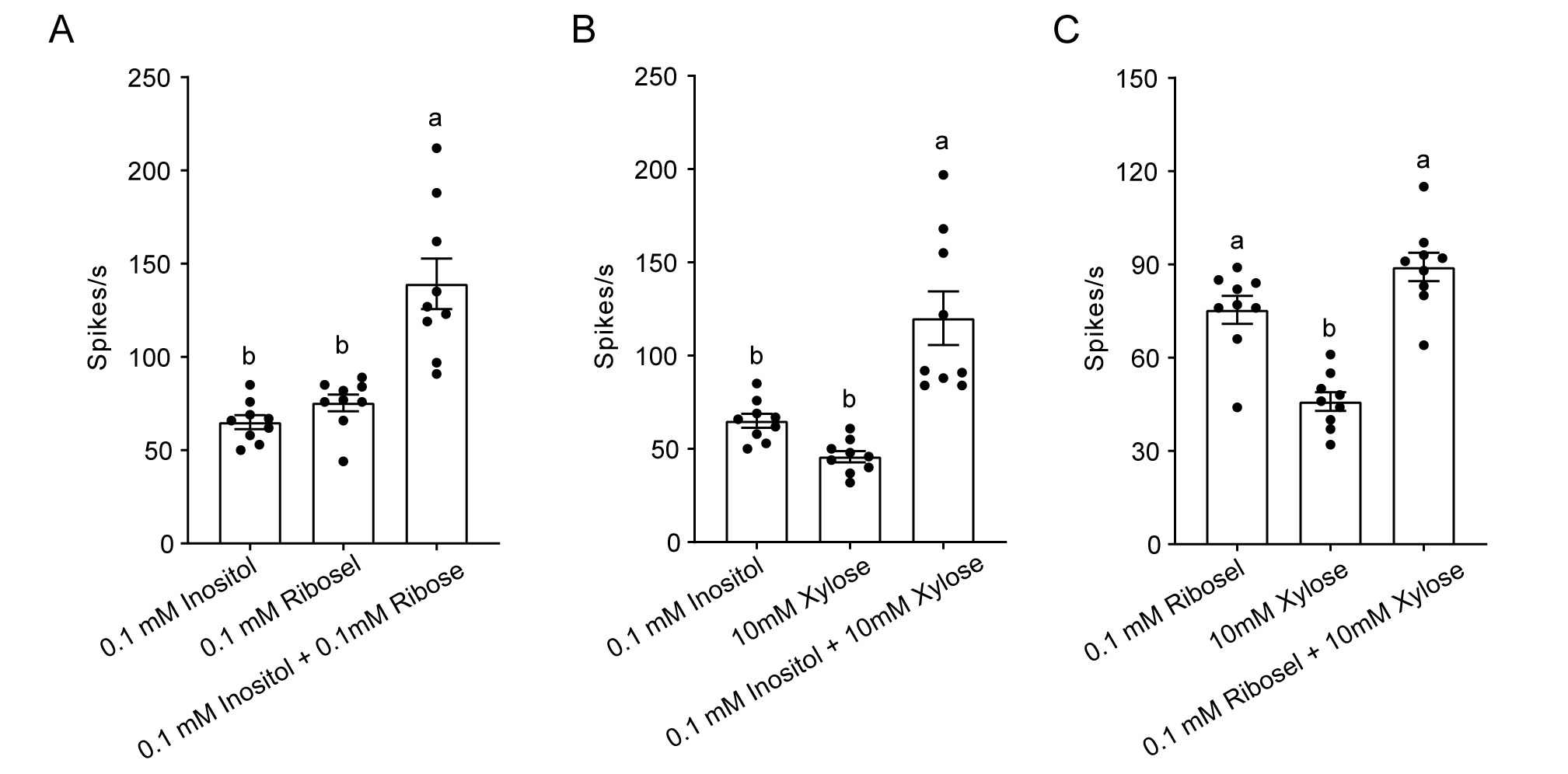

Supplement: S2 Fig — (A-C) Quantifications of firing rates of medial sensilla styloconica for 0.1 mM inositol, 0.1 mM ribose, and 10 mM xylose and their binary mixture (n = 9). Data are mean ± SEM. Data were analyzed by one-way ANOVA with Tukey’s HSD test (p < 0.05). (TIF) [file pgen.1011744.s002.tif]

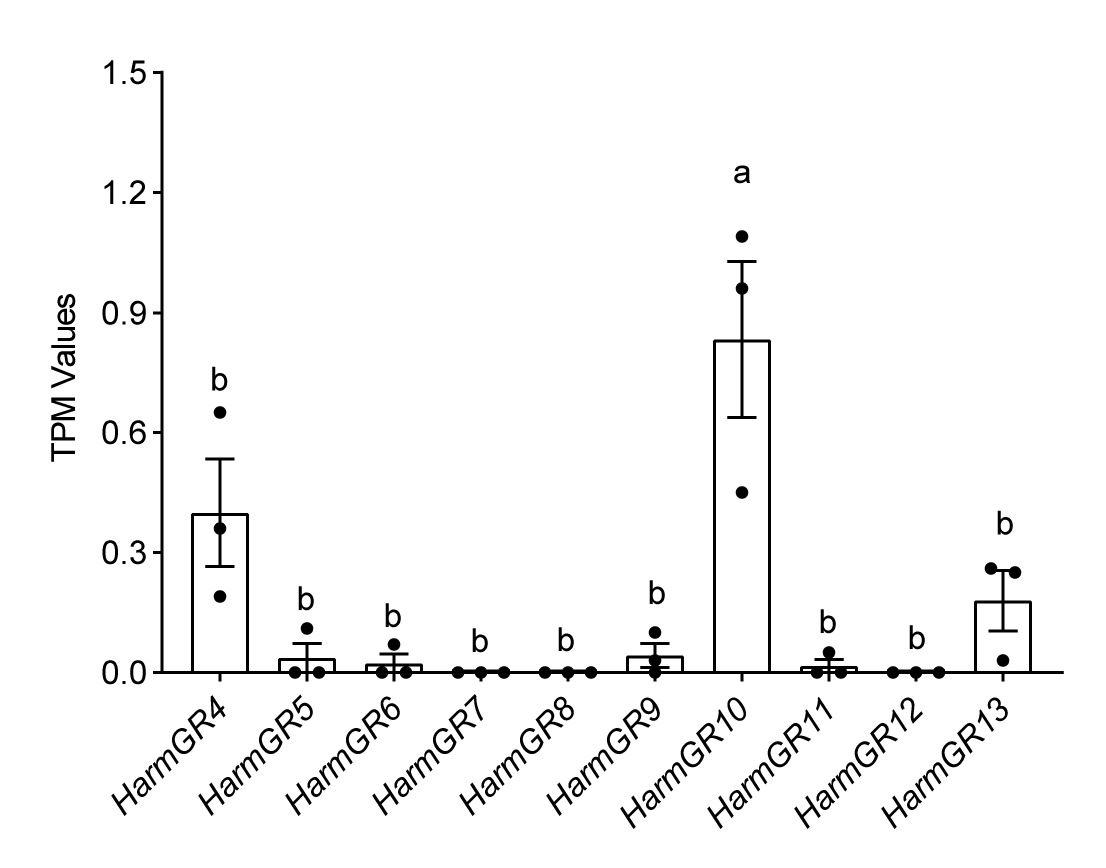

Supplement: S3 Fig — TPM values of 10 annotated sugar and fructose receptors obtained via transcriptome sequencing (n = 3). Data are mean ± SEM and analyzed by one-way ANOVA with Tukey’s HSD test (p < 0.05). (TIF) [file pgen.1011744.s003.tif]

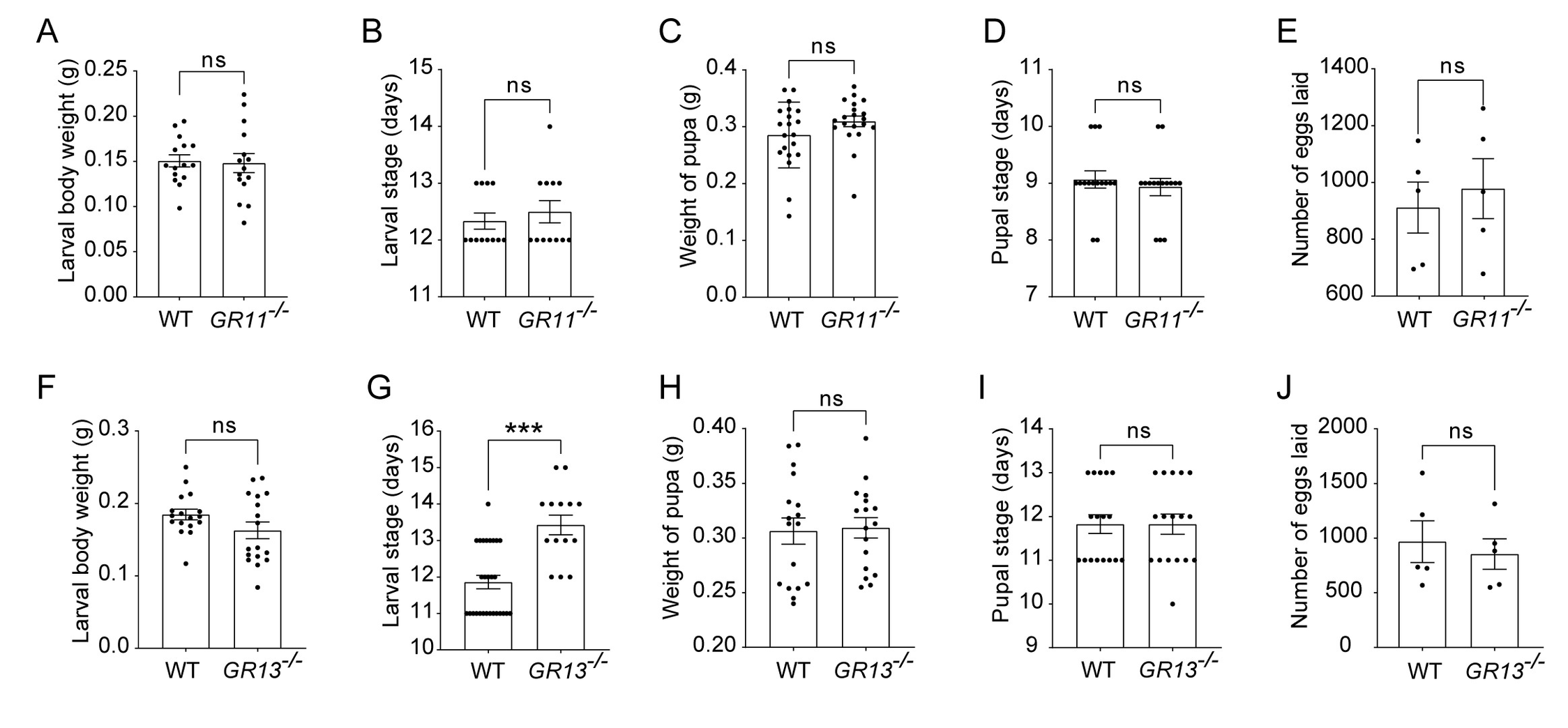

Supplement: S4 Fig — (A-E) Comparisons between GR13-/- and WT in larval weight, larval stage, pupal weight, pupal stage, and number of eggs laid. (F-J) Comparisons between GR11-/- and WT for the same parameters. A, C, D n = 17; B n = 27,14; E, J n = 5; G, I n = 15; G n = 12; H n = 20; (A–J) Data are mean ± SEM; ns, p > 0.05; *p < 0.05; **p < 0.01; ***p < 0.001. Data were analyzed by independent-samples t-tests. (TIF) [file pgen.1011744.s004.tif]

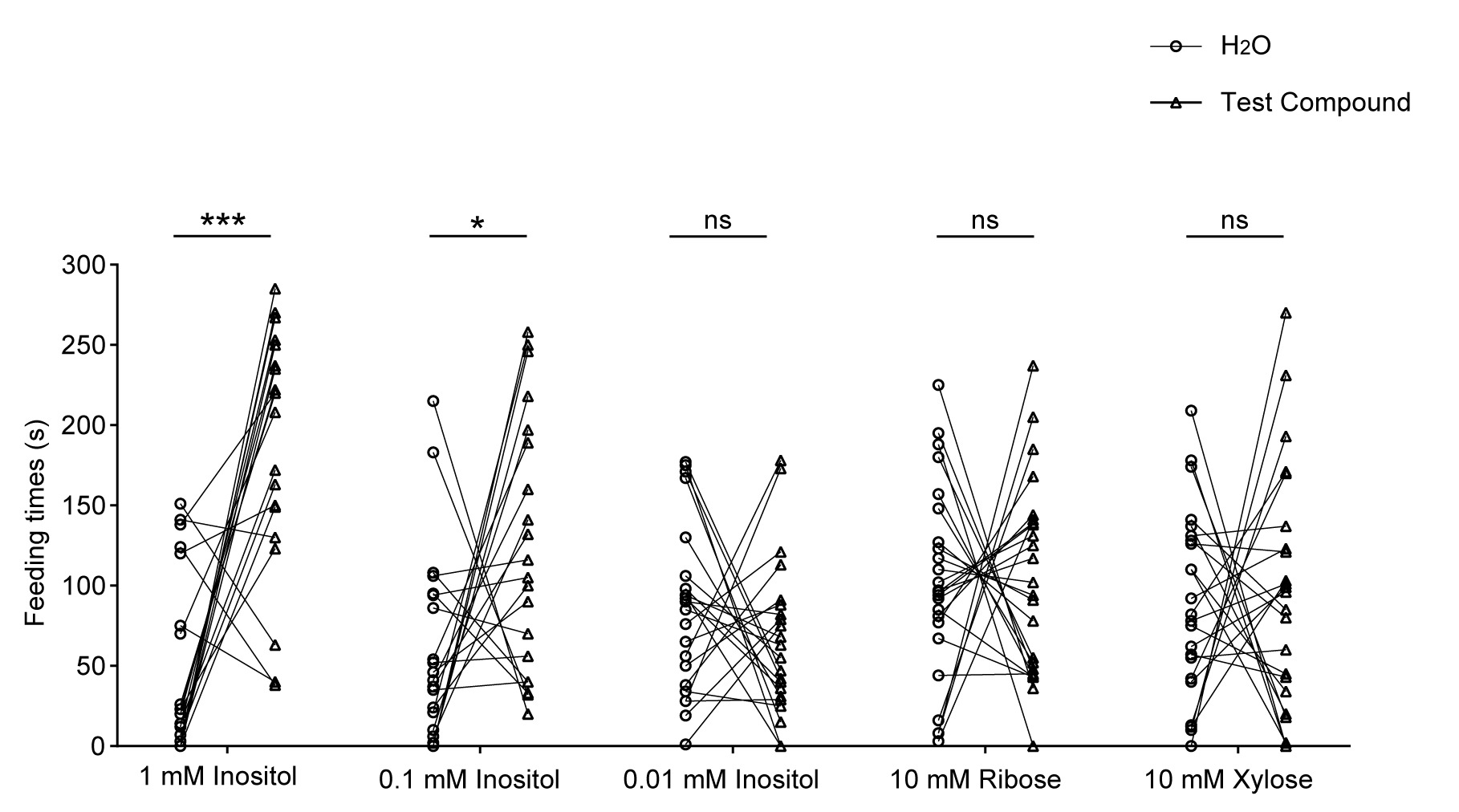

Supplement: S5 Fig — Feeding responses to inositol at 1 mM, 0.1 mM, and 0.01 mM (n = 19–21); ribose at 10 mM (n = 24); and xylose at 10 mM (n = 23). Each line represents one larva. *p < 0.05; **p < 0.01; ***p < 0.001. Data were analyzed by paired t-test. (TIF) [file pgen.1011744.s005.tif]

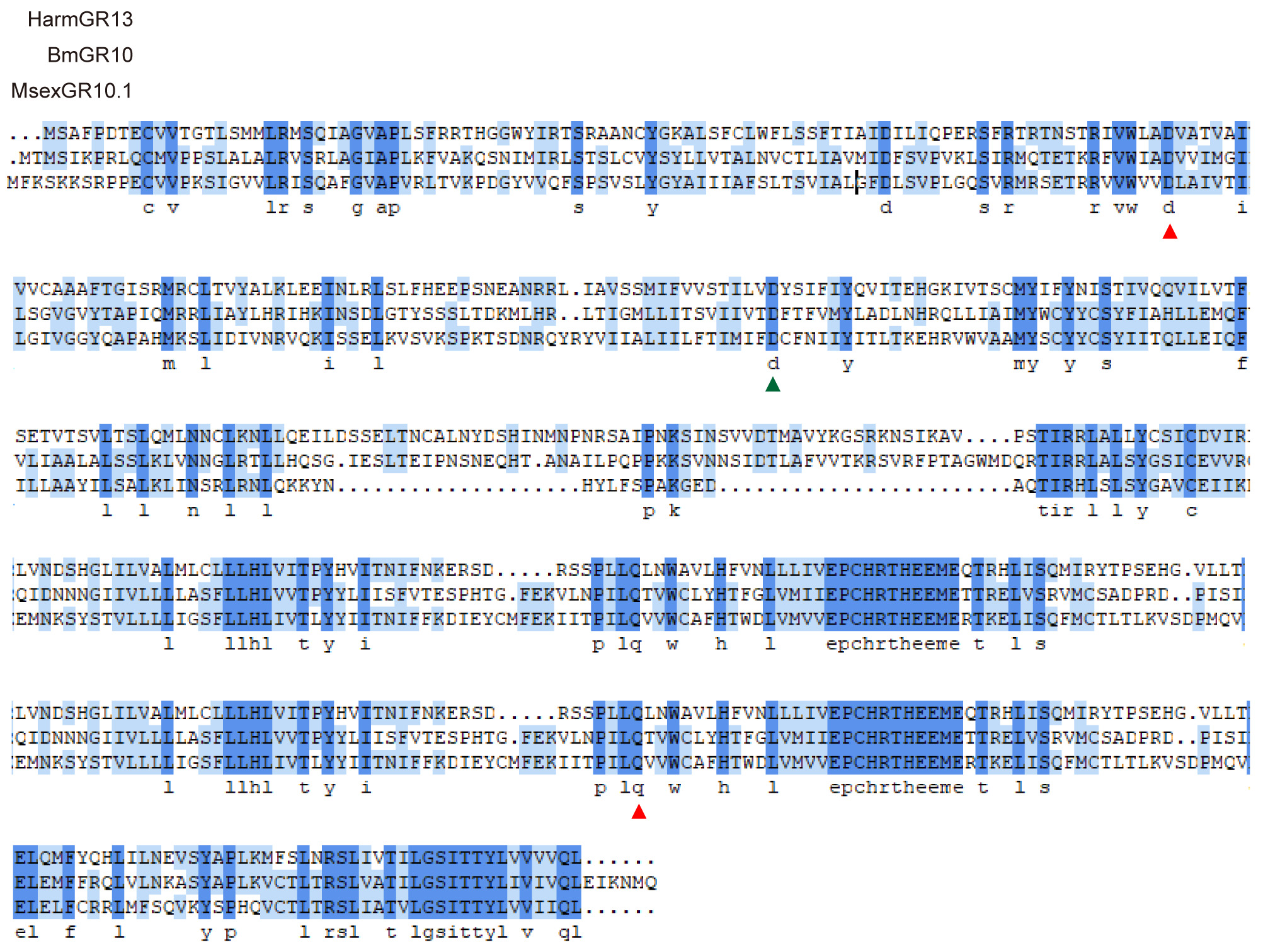

Supplement: S6 Fig — Arrows indicate key residues identified via molecular docking: D92, D159, and Q339. Red arrows denote residues involved in polar interactions with both inositol and ribose; green arrows denote residues interacting only with inositol. (TIF) [file pgen.1011744.s006.tif]
